# An Apparatus for Chloroform Oxygen Anæsthesia

**Published:** 1896-04

**Authors:** Llewellys Eliot

**Affiliations:** Washington, D. C.


ARTICLE VII.

### AN APPARATUS FOR CHLOROFORM-OXYGEN ANÆSTHESIA.

BY LLEWELLYS ELIOT, M. D., WASHINGTON, D. C.

It is not my intention to consider the relative merits of the various agents employed to produce anæsthesia for surgical purposes, nor to enter upon a discussion of the reasons for combining oxygen with the agent selected. These reasons will be sufficiently apparent to those who have employed chloroform or ether to any extent. My object in presenting the subject is simply to refer to the different kinds of apparatus that have been used.

According to the observations of those who have employed the combination of either chloroform-oxygen or ether-oxygen, the length of time necessary to induce anæsthesia varies from three to fifteen minutes, there is an absence of vomiting or nausea or headache which so often attend the usual anæsthesia,

The first article upon this combination which I saw was by Dr. Carter S. Cole in the "Medical Record," October 12, 1895. He describes his apparatus, which was the Wailon oxygen apparatus, with a few additions. The cone is a simple tin box shaped like a coffee can. In the middle of the top, a tin tube three inches long is obliquely inserted, half remaining outside. Two rubber tubes leave the bottle four-fifths full of ether, one of which fits on the tube in the top of the cone the other in the oxygen tank. The bottle used has been the "wash" bottle ordinarily employed in giving oxygen gas. An additional opening in the top of the cone, which is stoppered by a cork, provides for the addition of ether without removing the cone from the face. A perforated platform, held in place by a removable wire frame, keeps the gauze and cotton away from the patient's mouth, and a rubber mouth-piece that fits the cone and the patient's face completes the apparatus. The method first tried was to pass the oxygen through the regular wash bottle, then through the ether itself in another bottle, and thence through a tube to the cone, into which the gauze and cotton moistened with ether had been placed. Later the wash bottle was omitted, and the tube run from the oxygen tank to the bottle of ether, and the other tube direct to the cone, the gauze and cotton still being kept in the cone. Next the gauze and cotton were left out of the cone and the oxygenized ether passed into the cone. Dr. Cole states further that the amount of gas used probably averaged a gallon a minute. In the same article Dr. Francis H. Markoe describes his method of administering ether with oxygen, but the apparatus does not differ from Coles' except that he retains the two bottles.

Dr. J. H. McClelland describes ("N. A. Jour. Homœopathy, August, 1895), a method of administering chloroform with oxygen. It consists simply of forcing oxygen gently through a small body of chloroform. The apparatus consists of an ordinary chloroform inhaler, such as Krohne & Geseman's, of London, preferred, or a Junker. Instead, however, of forcing air through the chloroform bottle with a bulb, as ordinarily done, a tube is substituted, which is connected with a cylinder of oxygen. This is turned on fast or slow to suit the necessities of the case. The average amount of chloroform used is four drams an hour. The average time required to bring the patient under the influence is four and one-half minutes in a record of 100 cases. Albuminuria is no bar to its use.

Dr. H. L. Northrop, ("Hahnemannian Monthly," November, 1895), describes an apparatus which he has designed.

A nicely polished wooden box, 18 inches long, 7 inches wide, 7 inches deep, contains a steel cylinder holding 40 gallons of pure oxygen, a graduated two-ounce bottle, the requisite length of rubber tubing, and the inhaler. The latter has an inflatable face-shield attached to a metallic hood, with which is also connected a rubber respiratory bag. This, with the small wheel to be attached to the cylinder, the perforated rubber cork and nickel-plated brass tubes, is all the apparatus necessary for the proper administration of chloroform and oxygen. The apparatus weigh 19¾ pounds.

In order to use the apparatus put two ounces of pure chloroform into the bottle, attach the tubes to the cylinder and perforated cork, and place the bottle in the corner near the open end of the box, passing the tube leading to the inhaler through this opening. Put out the cylinder until it touches the bottle, and fix it in a position by tightening the screw in the top of the iron ring supporting the cylinder. Put the wheel on the cylinder valve, close and fasten the lid of the box, and the apparatus is ready.

The apparatus which I think the most satisfactory is one made by S. S. White Dental Company, which is a conversion of their ordinary oxygen apparatus to the purposes of the chloroform-oxygen or the ether-oxygen combination. It consists of a cylinder holding forty gallons of oxygen, a rubber tubing about six inches leading from the cylinder to the air bag, which holds one gallon, rubber tubing about ten inches leading from the air bag to the chloroform bottle, a rubber tubing of suitable length leading from the chloroform bottle to the inhaler. The face piece is of rubber, fit closely over the nose; and mouth, in the handle (a tube of hard rubber) is a stop-cock, so that the inhalation may be stopped instantly. When the air bag has been filled, the supply from the cylinder is shut off, and the inhalation begun; as the bag becomes empty, a turning of the wheel will again fill it. This can be continued so long as necessary. The patient breathes naturally, and the narcosis is relieved of much of its dangers. While this method of inducing anæsthesia is in its infancy, there are great possibilities in store for it in the near future. *Virginia Med. Monthly.*

